# A limited sampling schedule to estimate individual pharmacokinetics of pemetrexed in patients with varying renal functions

**DOI:** 10.1007/s00280-019-04006-x

**Published:** 2019-12-18

**Authors:** Nikki de Rouw, Sabine Visser, Stijn L. W. Koolen, Joachim G. J. V. Aerts, Michel M. van den Heuvel, Hieronymus J. Derijks, David M. Burger, Rob ter Heine

**Affiliations:** 1 Department of Pharmacy, Radboud Institute for Health Sciences, Radboud University Medical Center, P.O. Box 9101, 6500 HB Nijmegen, The Netherlands; 2 Department of Pharmacy, Jeroen Bosch Hospital, ‘s-Hertogenbosch, The Netherlands; 3 Department of Pulmonary Medicine, Amphia Hospital, Breda, The Netherlands; 4 Department of Pulmonary Medicine, Erasmus MC Cancer Institute, Rotterdam, The Netherlands; 5 Department of Medical Oncology, Erasmus MC Cancer Institute, Rotterdam, The Netherlands; 6 Department of Pharmacy, Erasmus MC, Rotterdam, The Netherlands; 7 Department of Pulmonology, Radboud Institute for Health Sciences, Radboud University Medical Center, Nijmegen, The Netherlands

**Keywords:** Pemetrexed, Limited sampling, Pharmacokinetics, TDM

## Abstract

**Purpose:**

Pemetrexed is a widely used cytostatic agent with an established exposure–response relationship. Although dosing is based on body surface area (BSA), large interindividual variability in pemetrexed plasma concentrations is observed. Therapeutic drug monitoring (TDM) can be a feasible strategy to reduce variability in specific cases leading to potentially optimized pemetrexed treatment. The aim of this study was to develop a limited sampling schedule (LSS) for the assessment of pemetrexed pharmacokinetics.

**Methods:**

Based on two real-life datasets, several limited sampling designs were evaluated on predicting clearance, using NONMEM, based on mean prediction error (MPE %) and normalized root mean squared error (NRMSE %). The predefined criteria for an acceptable LSS were: a maximum of four sampling time points within 8 h with an MPE and NRMSE ≤ 20%.

**Results:**

For an accurate estimation of clearance, only four samples in a convenient window of 8 h were required for accurate and precise prediction (MPE and NRMSE of 3.6% and 5.7% for dataset 1 and of 15.5% and 16.5% for dataset 2). A single sample at *t* = 24 h performed also within the criteria with MPE and NRMSE of 5.8% and 8.7% for dataset 1 and of 11.5% and 16.4% for dataset 2. Bias increased when patients had lower creatinine clearance.

**Conclusions:**

We presented two limited sampling designs for estimation of pemetrexed pharmacokinetics. Either one can be used based on preference and feasibility.

## Introduction

Pemetrexed is an anti-folate drug which is widely used as first and second-line treatment of non-small cell lung cancer and mesothelioma [1, 2]. There is a relationship between pemetrexed pharmacokinetics and toxicity [3–5]. Despite the introduction of prophylactic use of folic acid and vitamin B12 to reduce the risk of haematological toxicity, neutropenia remains a main exposure-related and treatment-limiting adverse reaction [3]. Latz et al*.* [3] showed that higher exposure relates to both decrease in neutrophil count and a longer recovery time after neutropenia.

Currently, pemetrexed is dosed based on body surface area (BSA) and this introduces large intraindividual variability in exposure [6]. There are several other factors which can contribute to variability in exposure, such as change in renal function or drug interactions [6-9]. Since pemetrexed exposure correlates well with toxicity [3, 10], pharmacokinetically (PK) guided dosing may be a feasible strategy to optimize treatment. Previously, the proposed target for safe and effective treatment is an AUC of 164 mg*h/L ± 25% [3, 6]. A prerequisite to validate this target for PK-guided dosing is the availability of an accurate, precise and clinically feasible limited sampling schedule (LSS) to assess the AUC.

From a patient’s perspective, a minimally invasive strategy is desired in a short time window. Therefore, our aim was to develop a LSS for the assessment of pemetrexed pharmacokinetics to use in clinical practice.

## Methods

### Limited sampling design evaluation

The predictive performance of several limited sampling designs to predict the pemetrexed clearance were evaluated. To assess individual exposure, the AUC can be calculated from clearance and the administered dose ($\mathrm{A}\mathrm{U}\mathrm{C}= \frac{\mathrm{D}\mathrm{o}\mathrm{s}\mathrm{e}}{\mathrm{C}\mathrm{l}\mathrm{e}\mathrm{a}\mathrm{r}\mathrm{a}\mathrm{n}\mathrm{c}\mathrm{e}}$). The previously developed and validated pharmacokinetic model by Latz et al. [3] was used to obtain the empirical Bayesian estimates for clearance using the post-hoc option in the software package NONMEM v7.4.3 [Icon, Ireland]. First, the full pharmacokinetic curves were fitted and obtained clearances were assumed to be ‘true values’. Subsequently, individual clearances were estimated from several limited sampling strategies based on the original dataset with certain timepoints removed.

The predictive performance was assessed with the mean relative prediction error (MPE %) for precision and normalized root mean squared error (NRMSE %) for accuracy, respectively. For MPE, confidence intervals were calculated as described by Sheiner et al*.* [11]. For NRMSE, relative uncertainty was determined according to the distribution-free approach of Faber [12]. Subsequently, corresponding confidence intervals were calculated.

Taking both patient’s perspective and statistical considerations into account, the pragmatical criteria for an acceptable LSS were defined as: a maximum of four sampling time points within 8 h with an MPE and NRMSE ≤ 20%. The value of acceptable precision, and, therefore, bias of clearance, depends on multiple factors such as expected analytical error, therapeutic range of the drug and the purpose of the LSS. For pemetrexed, we found this performance acceptable for the estimation of pemetrexed clearance.

## Datasets

Two separate datasets were used to evaluate several sampling designs. The first set contained pharmacokinetic data of 15 pemetrexed patients (from Visser et al*.* 2019) with adequate renal function (range creatinine clearance according to Cockcroft–Gault (CrCl–CG) 60–166 ml/min) [5]. Patients were treated according to label with a pemetrexed dose of 500 mg/m^2^ over a 10 min intravenous infusion. For dataset 1, the following sampling times were available 0.17–0.5–1–2–4–8–24 h after the start of administration. The second set included rich pharmacokinetic data of 47 individuals from JMAW phase I trial of Eli Lilly, with varying renal function (range CrCl–CG 17–200 ml/min.). These data were obtained through www.clinicalstudydatarequest.com [13]. The dose varied between patients but was administered over a 10 min intravenous infusion. The sampling times were 0.17–0.25–0.5–1–2–4–6–8–12–24–48–72 h after the start of administration. Since the used model of Latz et al. [3] was designed based on sampling up to 36 h after administration of pemetrexed, datapoints after 36 h were excluded from the analysis.

## Results

Table 1 presents the relevant baseline characteristics of the patients that were included in the two datasets and the results of the two best performing limited sampling designs. The second dataset contains patients with a wider range of both creatinine clearance and pemetrexed dose. For both datasets, several designs were tested based on the available sampling times. For an adequate estimation of pemetrexed clearance, within a sampling window of 8 h, four sampling times were required to reach acceptable precision and accuracy (MPE and NRMSE < 20%) in both datasets (not all data shown). As can be seen in Table 1, sampling at 0.5–2–4–8 h after administration resulted in an MPE and NRMSE of 3.6% and 5.7% for dataset 1. Using the second dataset, the performance of this sampling strategy was slightly lower but still within the acceptable range, with and MPE and NRMSE 15.5% and 16.5%, respectively. Table 1 also shows the performance of a single sample at *t* = 24 h. This strategy performed more or less equal to multiple sampling within 8 h, with imprecision and inaccuracy in the same order of magnitude. For all sampling designs, the MPE confidence interval did not include zero in both datasets, indicating a structural bias.

**Table 1:**
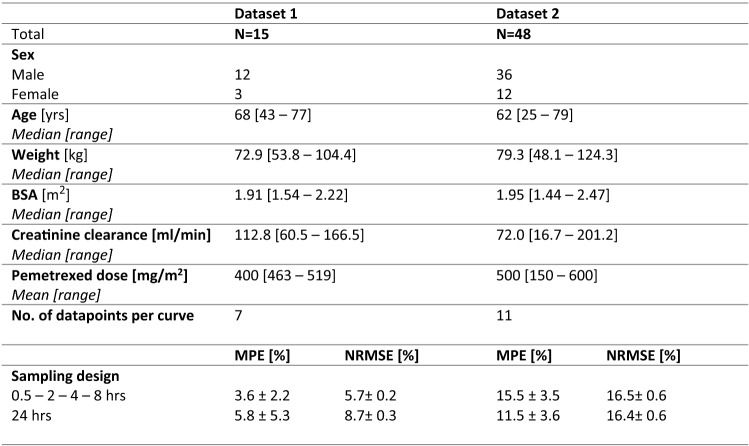
Baseline characteristics and predictive performance of best performing limited sampling designs

*MPE* mean percentage error, *NRMSE* normalized root mean squared error, *hrs* hours

**Fig. 1:**
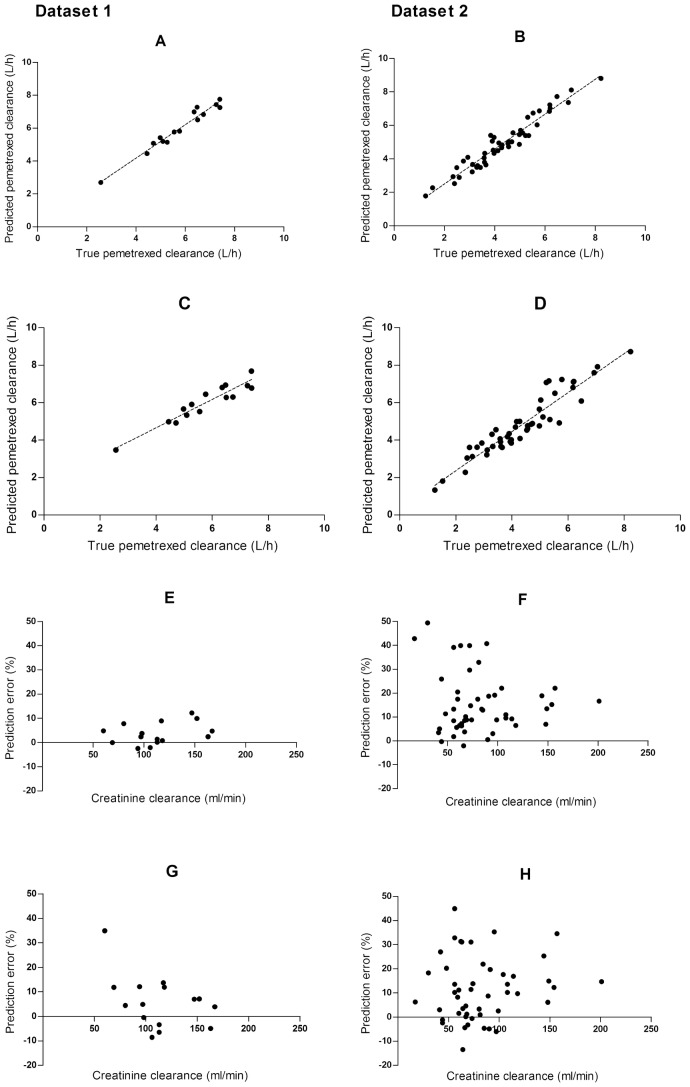
**a** True pemetrexed clearance versus predicted pemetrexed clearance for dataset 1, LSS 0.5–2–4–8 h. **b** True pemetrexed clearance versus predicted pemetrexed clearance for dataset 2, LSS 0.5–2–4–8 h. **c** True pemetrexed clearance versus predicted pemetrexed clearance for dataset 1, LSS 24rs. **d** True pemetrexed clearance versus predicted pemetrexed clearance for dataset 2, LSS 24 h. **e** Creatinine clearance versus relative prediction error for dataset 1, LSS 0.5–2–4–8 h. **f** Creatinine clearance versus relative prediction error for dataset 2, LSS 0.5–2–4–8 h. **g** Creatinine clearance versus relative prediction error for dataset 1, LSS 24 h. **h** Creatinine clearance versus relative prediction error for dataset 2, LSS 24 h

Figure 1a–d show true versus predicted pemetrexed clearance for the two proposed limited sampling designs. There is an acceptable correlation between the predicted and true clearances. Single sampling at *t* = 24 h for dataset 1 (see panel C) apparently introduces a slight overprediction of pemetrexed clearance. This is not observed in the second dataset. In Fig. 1e–h, creatinine clearances versus bias (MPE %) are visualized. Generally, for lower creatinine clearances in dataset 2, a larger prediction error (MPE %) was observed.

## Discussion

Our aim was to develop a patient-friendly limited sampling strategy for pemetrexed to assess the exposure in clinical practice and for research purposes. We found that two approaches resulted in the acceptable estimation of clearance (which serves as a proxy for the exposure). We propose two possible sampling schedules: the first consists of four sampling times at 0.5–2–4–8 h after pemetrexed administration. The second approach is a single sample at *t* = 24 h. These sampling schedules can be used for dose optimization and therapeutic drug monitoring, in specific cases as proposed earlier. Either one can be chosen based on preference and practical feasibility.

In general, the selected LSSs seemed to slightly overpredict clearance in both datasets and both sampling strategies. Overprediction of clearance could possibly result in unwarranted dose adjustments resulting in toxic exposure. However, taking the proposed target AUC of 164 mg*h/L ± 25% in mind, this structural overprediction is not considered relevant, because it is still well within the therapeutic range. Especially for dataset 2, bias increased with decreasing creatinine clearance. An explanation for the observation of increasing bias is that the used model of Latz et al. [3] was developed using patients with adequate renal function. In renal impairment, larger variability may be introduced, which is not observed in patients with adequate renal function. Also, with decreasing clearance, early datapoints in the pharmacokinetic curve become less informative. For dataset 2, removing the 8 h timepoint resulted in unacceptable loss of accuracy and precision. Additionally, the result of the *t* = 24 strategy in dataset 2 showed that at a later sampling time there may be less bias in patients with extremely low creatinine clearance. Altogether, a single sample at *t* = 24 may a feasible strategy for clinical practice, but it may require an extra hospital visit for the patient instead of a short prolongation of stay.

Our limited samplings strategy aimed to accurately predict pemetrexed AUC. Although Latz et al. have previously suggested that pharmacokinetically-guided dosing using the AUC may result in improved treatment [3], there is currently no conclusive evidence that the AUC is the best pharmacokinetic parameter to predict efficacy and toxicity. For example, the cytotoxicity of other drugs from the antifolate class, like methotrexate, is concentration threshold driven [14]. Prospective studies should confirm the utility of AUC-guided dosing before implementing this in clinical practice.

Altogether, we presented two patient-friendly and reliable limited sampling designs for estimation of pemetrexed pharmacokinetics. We are now using the 4-point LSS for development of personalized dosing strategies for pemetrexed in ongoing clinical studies [15–17].
